# Palmitate Diet-induced Loss of Cardiac Caveolin-3: A Novel Mechanism for Lipid-induced Contractile Dysfunction

**DOI:** 10.1371/journal.pone.0061369

**Published:** 2013-04-09

**Authors:** Catherine J. Knowles, Martina Cebova, Ilka M. Pinz

**Affiliations:** Maine Medical Center Research Institute, Maine Medical Center, Scarborough, Maine, United States of America; University of Tübingen, Germany

## Abstract

Obesity is associated with an increased risk of cardiomyopathy, and mechanisms linking the underlying risk and dietary factors are not well understood. We tested the hypothesis that dietary intake of saturated fat increases the levels of sphingolipids, namely ceramide and sphingomyelin in cardiac cell membranes that disrupt caveolae, specialized membrane micro-domains and important for cellular signaling. C57BL/6 mice were fed two high-fat diets: palmitate diet (21% total fat, 47% is palmitate), and MCT diet (21% medium-chain triglycerides, no palmitate). We established that high-palmitate feeding for 12 weeks leads to 40% and 50% increases in ceramide and sphingomyelin, respectively, in cellular membranes. Concomitant with sphingolipid accumulation, we observed a 40% reduction in systolic contractile performance. To explore the relationship of increased sphingolipids with caveolins, we analyzed caveolin protein levels and intracellular localization in isolated cardiomyocytes. In normal cardiomyocytes, caveolin-1 and caveolin-3 co-localize at the plasma membrane and the T-tubule system. However, mice maintained on palmitate lost 80% of caveolin-3, mainly from the T-tubule system. Mice maintained on MCT diet had a 90% reduction in caveolin-1. These data show that caveolin isoforms are sensitive to the lipid environment. These data are further supported by similar findings in human cardiac tissue samples from non-obese, obese, non-obese cardiomyopathic, and obese cardiomyopathic patients. To further elucidate the contractile dysfunction associated with the loss of caveolin-3, we determined the localization of the ryanodine receptor and found lower expression and loss of the striated appearance of this protein. We suggest that palmitate-induced loss of caveolin-3 results in cardiac contractile dysfunction via a defect in calcium-induced calcium release.

## Introduction

Lipotoxicity, the accumulation of lipids in non-adipose tissues due to increased plasma free fatty acids and triglycerides, has been well documented in type I and type II diabetes and in obesity [1]–[5]. The high incidence of hyperlipidemia in the U.S. population (30% of adults and unknown but growing numbers of children and adolescents) and its associated health challenges (coronary artery disease, hypertension, diabetes, heart failure, and stroke) make it imperative to understand the lipotoxic mechanisms that induce pathologic changes in tissues and organs. In particular, changes in cellular membrane lipid composition due to intracellular accumulation of lipids may have profound effects on membrane and organelle function. Several genetic rodent models recapitulate lipid accumulation in the heart, which is linked to lipid-induced cardiomyopathy [6]–[10].

To define the role of fatty acids in this process, previous studies have focused on the difference between the effects of saturated and unsaturated fatty acids on cardiac substrate utilization and the contractile performance of isolated cardiomyocytes [11], [12]. These studies show that exposing isolated cardiomyocytes to high concentrations of the saturated fatty acid palmitate induces contractile dysfunction and apoptosis [11], [13]–[15]. In contrast to these studies, we have focused on the effect of palmitate on the caveolae membrane system.

Caveolae are plasma membrane invaginations, 50–100 nm in diameter, rich in cholesterol and sphingolipids [16], and are important in endocytosis, lipid trafficking, and signal transduction [17], [18]. Originally discovered in the endothelium [19], caveolae have now been identified in many cell types including epithelium, adipocytes, fibroblasts, and smooth, skeletal, and cardiac muscle cells. Three caveolin proteins have been described: caveolin-1, caveolin-2, and caveolin-3. Caveolin-1 is ubiquitously expressed and is required for the formation of caveolae, while caveolin-3 is a muscle-specific caveolin that is predominantly expressed in skeletal and cardiac muscle. Caveolin-3 localizes not only to the plasma membrane but also to T-tubules and is essential for normal formation of the T-tubule system in the developing muscle and heart [20], [21]. Consequently, the loss of caveolin-3 in the mouse reduces the number of caveolae in muscle and induces T-tubule abnormalities and produces a phenotype similar to muscular dystrophy [22], [23]. Loss of caveolin-1 or caveolin-3 in the heart induces hypertrophy that leads to dilated cardiomyopathy [24]–[27]. Caveolin-3 potentially also has an important role in cardiac calcium regulation [28], [29]. Therefore, we tested the hypothesis that dietary palmitate intake, via accumulation of membrane sphingolipids, induces the loss of caveolin-3 and causes cardiac contractile dysfunction. We also tested whether the dietary induced loss of caveolin-3 is reversible by normalizing the dietary intake of fat.

## Materials and Methods

### Human left ventricular tissue

Embedded tissue blocks of autopsy material for a total of 5 non-obese normal, 4 obese with no cardiomyopathy, 9 obese with cardiomyopathy, and 7 non-obese with cardiomyopathy cases with non-identifying information were obtained from the Maine Medical Center BioBank and underwent immunohistochemistry staining for caveolin-3 in the histology core of MMCRI. Patients with a body mass index of >30 were considered obese. Exemption for inclusion of these tissue samples in this study was obtained from the IRB at MMCRI.

### Animals

C57BL/6 mice of both genders were used. The age range covered by this study was 15–16 weeks for the 12-week feeding time point and 27–28 weeks for the 24-week time point. The Maine Medical Center Institutional Animal Care and Use Committee approved all experimental protocols. The handling and housing of mice followed the recommendations of current NIH and American Physiological Society guidelines for the care and use of laboratory animals.

### Diet composition and feeding regimen

High lipid diets were custom made by Harlan Laboratories. All high fat diets contained 200 g/kg as fat (20%). In the high palmitate diet 90 g/kg was palmitate, equivalent to 41% of all fat being palmitate (Harlan TD05235) and fed ad libitum. The rationale for choosing this lipid composition is based on the average saturated fat content of a fast food meal and taking into consideration that the saturated fat content of hamburgers can be more than 30%. As control for the high fat content a diet was composed using medium chain triglycerides with the same caloric intake from fat as the high palmitate diet (Harlan TD05237). Standard laboratory chow was used as comparison with a crude fat content of 6%. Mice were changed to high fat diets at the age of 3–4 weeks and maintained for 12 weeks. After 12 weeks they underwent cardiac function assessment and a subset of mice were returned to normal standard laboratory chow for another 12 weeks. At the end of contractile function assessment, tissues were frozen and used for either total lipid analysis or caveolin protein determination. A subset of mice was used for isolating cardiomyocytes to determine intracellular localization of caveolin isoforms by confocal microscopy.

### Blood plasma biochemistry

Blood plasma samples were obtained biweekly by cheek pouch bleeding. Glucose, ketones, cholesterol, HDL, and triglycerides levels in whole blood were determined using a CardioChek Analyzer (Polymer Technology Systems, Inc. Indianapolis, IN). Insulin levels were measured using the ultrasensitive ELISA kit from ALPCO Diagnostics, Salem, NH.

### Langendorff perfusion

Mice were anesthetized using avertin and the heart perfused following Pinz et al. [30]. Briefly: After cervical dislocation, the heart was quickly excised and arrested in ice-cold Krebs Henseleit buffer (in mM: EDTA 0.5, NaCl 118, KCl 5.3, Mg_2_SO_4_ 1.2, NaHCO_3_ 25, CaCl_2_ 2.5, glucose 10, pyruvate 0.5). Then, the heart was hung by the aorta to a cannula and perfused at a constant pressure of 75 mmHg. A water-filled balloon was inserted into the left ventricle via the left atrium and, by inflation, the end diastolic pressure was adjusted to 5–10 mmHg. The balloon line was connected to a pressure transducer and pressure changes in the left ventricle were followed on line with a PowerLab data acquisition system. To exclude differences in contractile performance due to varying heart rates, hearts were paced at 420 bpm throughout the protocol. Contractile performance was assessed in the presence of different extra-cellular calcium concentrations (1.5–4.0 mM) to determine contractile reserve with increasing cardiac workload and to define cardiac calcium sensitivity.

### Isolation of adult mouse cardiomyocytes

Adult mouse cardiomyocytes were isolated following Pinz et al. [31]. Briefly, mouse hearts were perfused on a gravity flow system with Tyrode's solution containing calcium to clear coronaries of blood. Then calcium-free Tyrode's solution was used to initiate the digestion protocol. The enzymatic digestion of heart tissue was accomplished by using Liberase TH (Roche). Liberase TH solution was flushed from the heart tissue after 4–5 minutes with calcium-free Tyrode's solution, and then cardiomyocytes were mechanically dispersed by gently teasing apart tissue. Cells were re-adjusted to 1.2 mM extracellular calcium and were plated on mouse laminin coated cover slips. Only freshly isolated cells were used and cells were fixed the same day.

### Confocal microscopy

After adhesion to laminin coated cover slips, cardiomyocytes were washed twice in ice cold PBS and fixed with 4% paraformaldehyde in PBS for 10 minutes at RT. Coverslips were washed with ice cold PBS and stored in PBS with 0.01% sodium azide. Fixed cells were blocked and permeabilized with 5% BSA, 0.1% Triton-X-100, 0.1% Tween20, and 0.1% sodium azide in PBS for 1 hour at RT. Primary antibodies were diluted in PBS with 1% BSA and 0.3% Triton-X-100: Caveolin-1 (Cell Signaling #3267, 1∶200), Caveolin-3 (BD Transduction #610420, 1∶300). Primary antibodies were incubated at 4°C overnight prior to conjugation with fluorescently labeled secondary antibodies. Alexa Fluor 488 labeled goat anti-mouse IgG 1∶2000 (Invitrogen) and Alexa Fluor 546 goat anti-mouse IgG 1∶2000 (Invitrogen) were incubated for 2 hours at RT. Counter staining with a DNA stain TOPRO (Invitrogen T3605, 1∶1000) was completed for 30 minutes at RT directly after secondary antibody incubation. Cells were mounted with ProLong Gold anti-fade reagent (Invitrogen), allowed to cure overnight at RT, sealed and examined with a Leica TCS SP II True Confocal Laser Scanning Microscope and analyzed using the Leica confocal software.

### Lipid extraction, fractionation, and mass spectrometry

ESI/MS/MS analysis of endogenous sphingosine bases, sphingoid base-1-phosphates, ceramide, and of endogenous sphingomyelin molecular species were performed on a Thermo-Fisher TSQ Quantum triple quadrupole mass spectrometer, operating in a Multiple Reaction Monitoring (MRM) positive ionization mode, using modified version [32]. Briefly, for ceramide species, cardiac tissue extracts corresponding to 1 mg protein, were fortified with the internal standards (ISs: C_17_ base D-erythro-sphingosine (17CSph), C_17_ sphingosine-1-phosphate (17CSph-1P), N-palmitoyl-D-erythro-C_13_ sphingosine (13C16-Cer) and heptadecanoyl-D-erythro-sphingosine (C17-Cer)), and extracted with ethyl acetate/isopropanol/water (60/30/10 v/v) solvent system. After evaporation and reconstitution in 100 µl of methanol samples were injected on the HP1100/TSQ Quantum LC/MS system and gradient eluted from the BDS Hypersil C8, 150×3.2 mm, 3 µm particle size column, with 1.0 mM methanol ammonium formate/2 mM aqueous ammonium formate mobile phase system. Peaks corresponding to the target analytes and internal standards were collected and processed using the Xcalibur software system. For sphingolipids, cardiac tissue extracts (1 mg protein), were fortified with the internal standards (IS): N-hexanoyl-1-(2-phosphorylcholine)-sphingosine (C6-SM), N-heptadecanoyl-1-(2-phosphorylcholine)-sphingosine (C17-SM) and extracted with ethyl acetate/iso-propanol/water (60/30/10%v/v) solvent system. After evaporation and reconstitution in 1 ml of methanol 10 µl of 1M NaOH in methanol was added, then left for 2 hours at room temperature, and then subjected to Bligh & Dyer extraction (methanol/chloroform/water). The lower, organic phase was recovered, evaporated to dryness, and reconstituted in 200 µl of methanol. The reconstituted samples (10 µl) were injected into the LC/MS and analyzed as described above. Quantitative analysis for ceramide and sphingomyelin species was based on the calibration curves generated by introducing an artificial matrix with the known amounts of target analyte synthetic standards and an equal amount of the internal standards (ISs). The target analyte/IS peak areas ratios were plotted against analyte concentration. The target analyte/IS peak area ratios from the samples were similarly normalized to their respective ISs and compared to the calibration curves, using a linear regression model.

### Statistics

Sphingolipid metabolites were analyzed with two-way ANOVA and Bonferroni post-hoc test, cardiac contractile function data were analyzed with linear regression analysis in GraphPad Prism (Prism 4, GraphPad Software, Inc.). All other data were analyzed with one-way ANOVA and p<0.05 was considered significant. Data are represented as mean ± SE.

## Results

### General characteristics of mice fed high fat diets

C57BL/6 mice (both genders) were maintained on standard, MCT or palmitate diet starting at 3 weeks of age for 12 weeks. To follow the change in blood lipid, glucose and insulin levels, blood samples were taken every two weeks. Over the time frame of this study there were no changes in blood parameters measured at any time points between diets: (all in mM, glucose: 5.8±0.8 standard, 5.4±0.4 MCT, 6.9±0.5 palmitate; ketones: 0.4±0.05 standard, 0.5±0.04 MCT, 0.5±0.02 palmitate; cholesterol: <2.59±0 standard, 3.6±0.1 MCT, 3.8±0.3 palmitate; HDL: 1.0±0.3 standard, <0.57±0 MCT, <0.57±0 palmitate; triglycerides: 1.0±0.3 standard, <0.57±0 MCT, <0.57±0 palmitate; n = 9). The only exception were insulin levels, which increased after 10 weeks in mice maintained on the palmitate diet, which reached significance only at the 12-week time point: (insulin (nM): 0.3±0.004 standard, 0.4±0.05 MCT, 1.9±0.3* palmitate; n = 6, * p<0.05, one-way ANOVA, Student Newman Keul's posthoc comparison.). On the high fat control diet insulin levels remained normal. Thus, mice remained normoglycemic and normolipidemic on either high fat diet, but were slightly hyperinsulinemic on the high palmitate diet for the duration of ∼2 weeks before the endpoint of the study.

To determine whether feeding a high palmitate diet induces hypertrophic changes in the heart, mice were weighed weekly and heart and left ventricular (LV) weight were determined after 12 weeks (Table 1). Mice maintained on the palmitate diet gained slightly more body weight, however, no significant differences were observed between diets (Table 1). Balloon volumes, which are a measure of LV volume, were unchanged in all groups. Thus, there is no indication of morphological changes, such as hypertrophy, associated with the high fat diets chosen for this study.

**Table 1:** General characteristics of mice at 12 weeks of the feeding regimen.

	standard	MCT	palmitate
n	5 (3m,2f)	7 (3m,4f)	5 (3m, 2 f)
BW (g)	27.2±4.7	25.9±4.1	30.7±6.1
LV (mg)	91±13.7	77±17	92±3.7
Bal vol. (µl)	12.3±4	10.4±3.7	9.4±2.2
HW (mg)	117±16	101±19	119±6
HW/BW (mg/g)	4.2±0.2	4.8±0.3	4.0±0.7

BW: body weight, HW: heart weight, Bal Vol.: balloon volume  =  left ventricular volume, LV: left ventricle, m: male, f: female. Means ± SE, n = 5–7.

### Palmitate-induced ceramide and sphingomyelin accumulation in cardiac tissue

**Figure 1:**
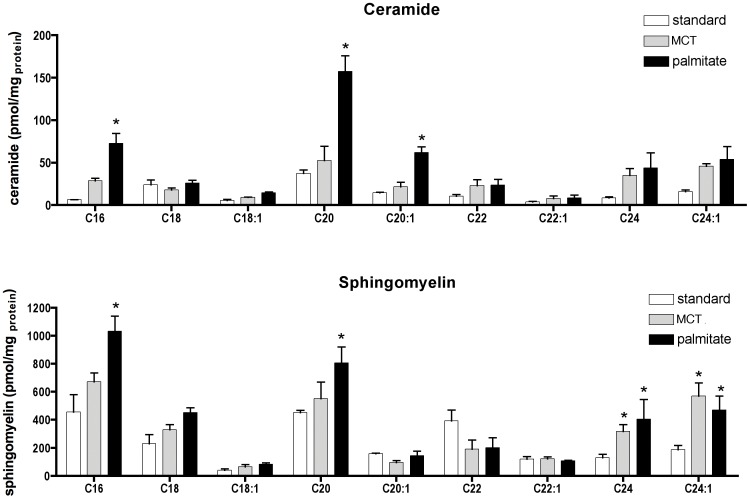
Palmitate increases medium and long chain ceramide and sphingomyelin species in left ventricular tissue. **A**) Levels of different ceramide species in total lipid extracts of left ventricular tissue determined by mass spectrometry after 12 weeks on standard, control or palmitate diet. Medium chain ceramides with C16, C20 and C20∶1 increase in mice fed palmitate for 12 weeks. Long chain ceramides C24 and C24∶1 are equally increased in control and palmitate diet fed mice, however these data did not reach significance. Mean ± SE, n = 3, two-way ANOVA, Bonferroni post-hoc analysis, * p<0.05. **B**) Sphingomyelin species levels at the 12 week feeding time point. Palmitate feeding led to an increase of medium chain sphingomyelin species C16 and C20. The long chain sphingomyelins, C24 and C24∶1, are equally increased in mice fed control or palmitate diet compared with standard diet fed mice. Mean ± SE, n = 3 (2 male, 1female each group), two-way ANOVA, Bonferroni post-hoc analysis, * p<0.05.

Ceramide and sphingomyelin species were determined in total lipid extracts of cardiac tissue after 12 weeks on the diets. Total ceramide levels (calculated as the sum of ceramide species) were significantly increased in palmitate diet fed mice with 393±19.4 pmol/mg protein (p = 0.03 vs. standard diet, one- way ANOVA) compared to 129±6.3 pmol/mg protein and 295±14.3 pmol/mg protein in standard or control diet fed mice. Palmitate feeding preferentially caused the accumulation of medium- and long chain (C16, C20 and C20∶1) ceramide species (Fig. 1A). Interestingly C18 did not increase, which is the ceramide species generated from de-novo synthesis from palmitate. This may suggest that there is an immediate deacetylation of C18 to C16 or an immediate acetylation to C20. The long chain ceramides from C22 to C24 did not show significant differences between standard, MCT or high palmitate diets. Other ceramide species were determined but data are not shown because of the low levels present.

The palmitate-induced accumulation of sphingomyelin did not reach significance for total sphingomyelin with 2170±308 pmol/mg protein in standard, 2284±401 pmol/mg protein in MCT, and 2837±71 pmol/mg protein in palmitate diet fed mice. However, there were significant changes in the accumulation of medium- and long chain length species, C16 and C20, between palmitate and standard or MCT diet fed mice (Fig. 1B). This result suggests that sphingomyelin synthase may limit the accumulation of medium chain ceramides by increasing the membrane content of medium chain sphingomyelins. Long chain sphingomyelins, C24 and C24∶1, were significantly increased in the two high fat diets compared to standard diet.

These changes in sphingolipid levels cannot be achieved using synthetic ceramide or sphingomyelin species as only short chain species (C2–C6) reliably cross the plasma membrane. Short chain ceramides and sphingomyelins do not occur at physiological levels but have been used to investigate the mechanism of ceramide-induced apoptosis in cell culture [33].

### Palmitate-induced systolic dysfunction after 12 weeks

**Figure 2:**
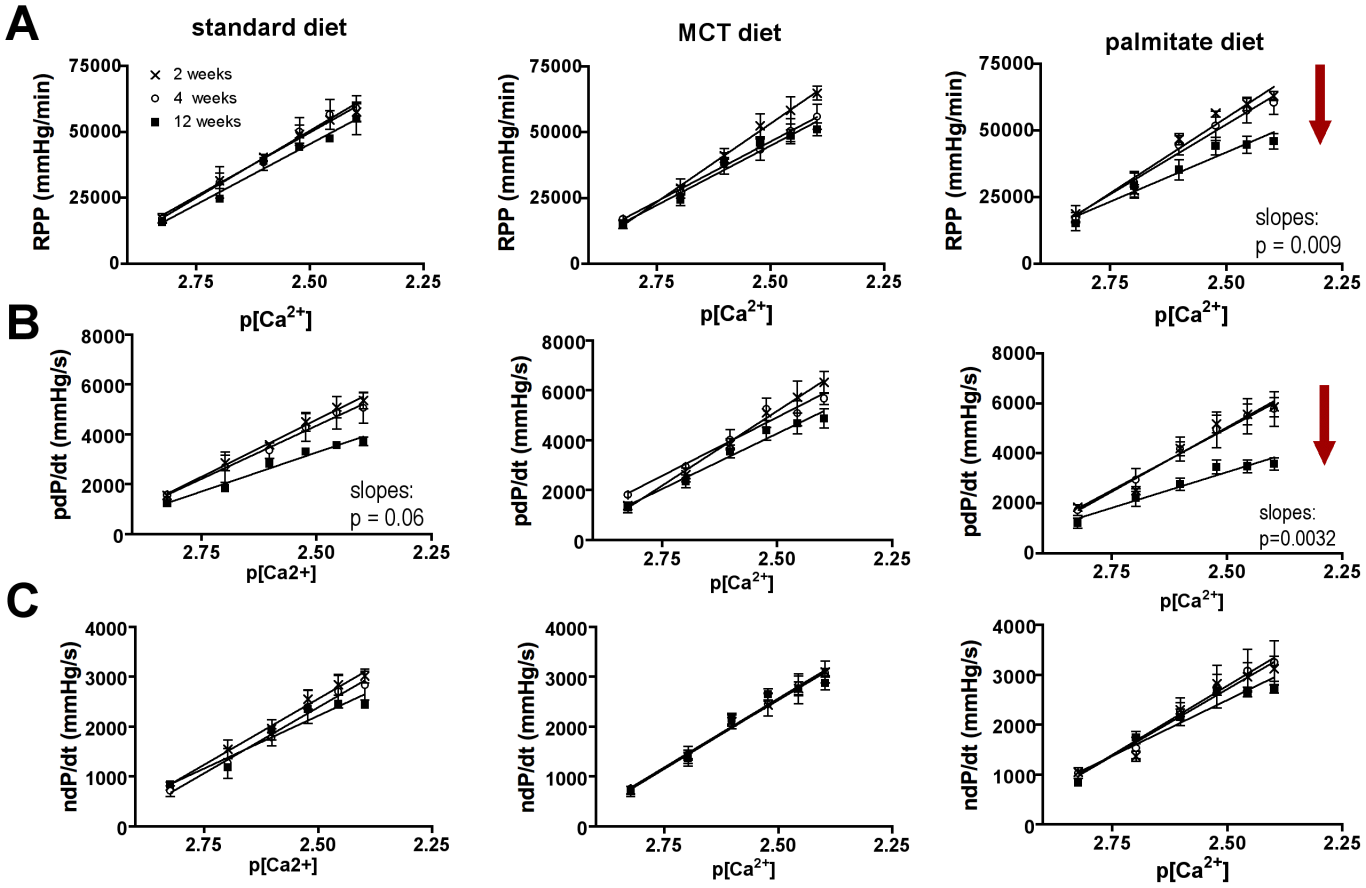
Palmitate diet causes systolic dysfunction in 12 weeks. **A**) Rate pressure product (RPP) vs. p[Ca^2+^] relationship for mouse hearts at 2 (x), 4 (○), and 12 (▪) weeks of standard, MCT, and palmitate diet. RPP decreased by 30% in mice after 12 weeks on the palmitate diet. Means ± SE, n = 4–8, linear regression analysis through single data points using GraphPad Prism 4. **B**) pdP/dt vs. p[Ca^2+^] relationship of mice fed standard, MCT, and palmitate diet for 2, 4, or 12 weeks. pdP/dt is decreased by 40% in mice fed palmitate diet for 12 weeks. Means ± SE, n = 4–8, linear regression analysis through single data points using GraphPad Prism 4. **C**) ndP/dt vs. p[Ca^2+^] relationship for all diets over 2, 4 and 12 weeks. No differences were observed in diastolic contractile performance. Means ± SE, n = 4–8 (50/50 gender split in all groups), linear regression analysis through single data points using GraphPad Prism 4.

Hearts of mice fed standard or MCT control diets had normal contractile performance, as indicated by the essentially equivalent calcium response curves at the 2, 4, and 12-week intervals (Fig. 2A–C). In contrast, after 12 weeks on the palmitate diet, mouse hearts showed systolic contractile dysfunction. Fig. 2A shows a 30% reduction in rate pressure product (RPP, a measure of the contractile work the heart performs) in high-palmitate-fed mice at 12 weeks. Positive dP/dt, the first derivative of pressure loss, a measure of contractility, showed a 40% reduction at 12 weeks (Fig. 2B). Maximum systolic pressure and developed pressure were similarly affected (data not shown). The contractile dysfunction was especially apparent at higher extracellular calcium concentrations, suggesting diminished calcium handling ability in these hearts. The diminished positive dP/dt indicates a slower release of calcium from the SR, which suggests that either the dihydropyridine receptor (DHPR, L-type calcium channel) or the ryanodine receptor (RyR) or both are affected. The DHPR localizes to caveolae, and the extradiadic RyR is in close proximity to caveolae [29].

**Figure 3:**
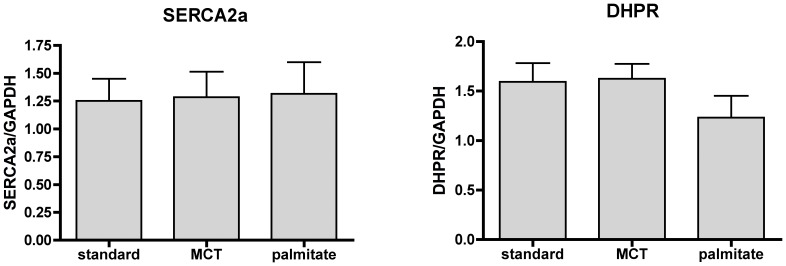
Palmitate diet does not change SERCA2a or DHPR protein levels. Quantification of SERCA (**A**) and DHPR (**B**) protein levels in LV tissue from hearts at the 12-week feeding time point. Protein levels were normalized to GAPDH levels. Both proteins are unchanged by the high fat feeding regimen. Means ± SE, n = 6–9 (3male and 3 female for n = 6, or 5 male and 4 female for n = 9).

The sarcoendoplasmic reticulum calcium ATPase (SERCA2a) is a major calcium handling protein, and changes in its activity and expression levels are known to cause contractile dysfunction in heart failure [34]. We tested whether SERCA2A or DHPR levels were changed due to the palmitate diet. However, protein levels for both proteins were the same in all diets (Fig. 3) and data did not show a trend for diminished expression under any treatment conditions.

### Palmitate-induced loss of T-tubular caveolin-3 and loss of striated RyR appearance

**Figure 4:**
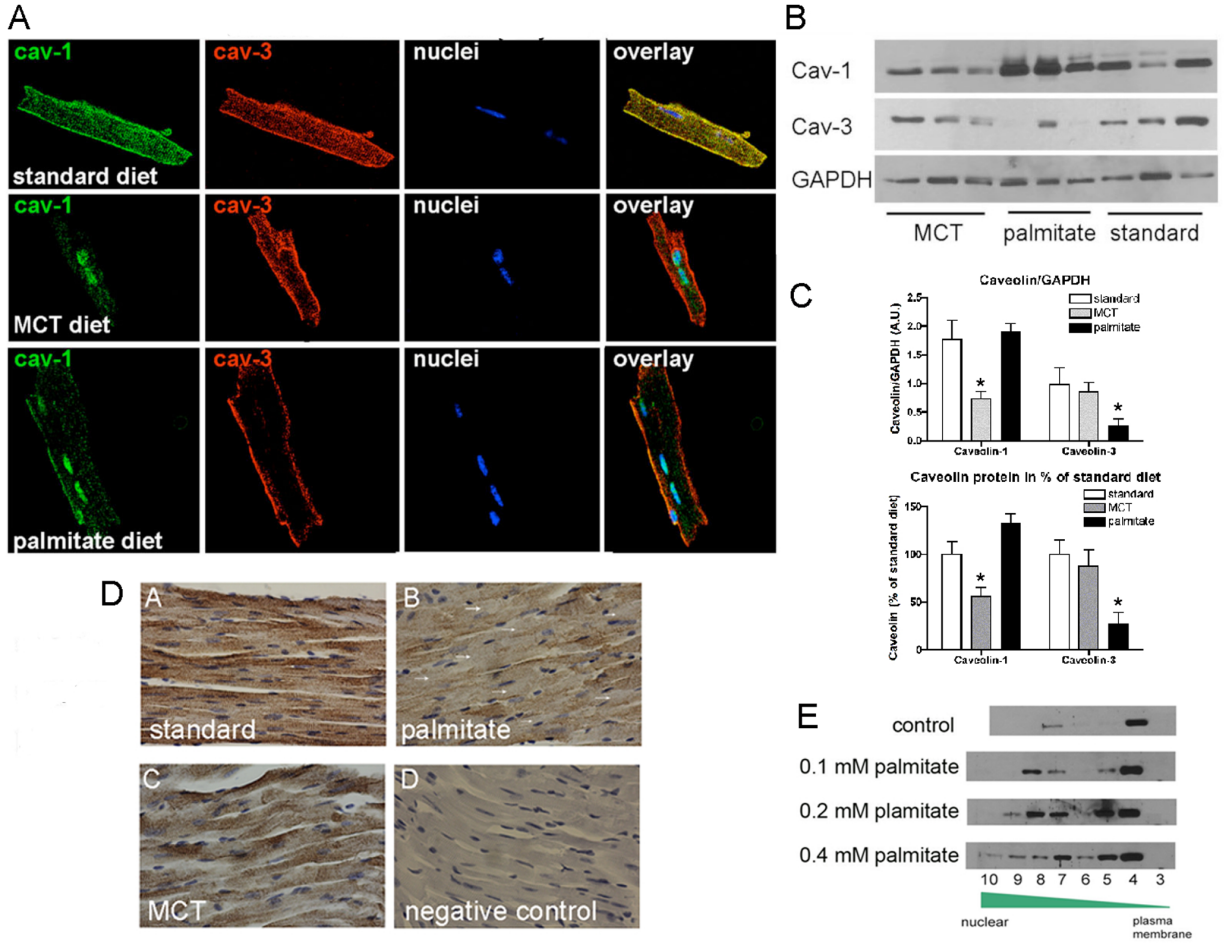
Palmitate-induced loss of caveolin-3 from the T-tubule system in the heart. **A**) Localization of caveolin-1 and -3 in confocal images of isolated cardiomyocytes from mice fed standard, MCT or palmitate diet for 12 weeks. Both caveolins co-localize in cardiomyocytes from standard diet fed mice, whereas caveolin-1 decreases in MCT diet fed mice and caveolin-3 is lost from the T-tubule system in palmitate diet fed mice. **B**) Western blot for caveolin-1 and -3 for all diets. **C**) Quantification of Western blots using GAPDH as loading control (top panel) and percent changes in the lower panel. N = 5 (3 male, 2 female), * p<0.05 one-way ANOVA. **D**) Representative images of immunohistochemistry for caveolin-3 in left ventricular tissue sections of standard, MCT, and palmitate diet fed mice. Caveolin-3 staining is observed at the plasma membrane and the T-tubule system in standard diet fed mice. The overall signal intensity is modestly decreased in MCT diet fed mice, but caveolin-3 also localizes to the plasma membrane and the T-tubule system. In palmitate diet-fed mice there is significant loss of caveolin-3 staining. Some cardiomyocytes do not show any staining for caveolin-3 (white arrows) at the plasma membrane or the T-tubules. N = 4 per diet. **E**) Cellular membrane fractionations by sucrose gradient centrifugation. HL-1 cardiomyocytes were exposed to increasing concentrations of BSA-complexed palmitate (0.1, 0.2, and 0.4 mM) and then underwent sucrose gradient centrifugation to separate the different membrane systems of the cells. By convention fraction 4 is considered the high buoyant, caveolae fraction and with increasing fraction number, buoyancy decreases and membranes become denser. Exposure to palmitate causes caveolin-3 to move from the caveolae fraction (fraction 4) to the denser membrane fractions (fraction 8–10), which represent SR, ER, and nuclear membranes. Experiment repeated 4 times with similar results.

To further investigate the mechanism of palmitate-induced contractile dysfunction, we isolated cardiomyocytes from all groups of mice and determined the intracellular localization of caveolin proteins and their expression levels. Caveolin-1 and caveolin-3 are expressed in cardiomyocytes of standard diet fed mice and co-localize to the plasma membrane and the T-tubule system (Fig. 4A). The MCT control diet decreased caveolin-1 protein levels and some signal was observed in the nuclei. The observation that caveolin-1 can localize to the nucleus under these conditions is surprising, and it remains an open question whether this state represents a distinct signaling function. Caveolin-3 levels and intracellular localization did not change with MCT diet feeding (Fig. 4A and B). In contrast, with high palmitate feeding, caveolin-3 is lost from the T-tubule system and from the plasma membrane, though diminished protein staining remains localized to the plasma membrane (Fig 4A). The expression of caveolin-3 decreases by 90% compared to standard diet-fed mice (Fig. 4 A–C). In addition, there was a modest increase in caveolin-1 expression during palmitate feeding with unchanged intracellular localization (Fig. 4C). To support our findings with confocal microscopy, left ventricular tissue sections underwent immunohistochemistry and were stained for caveolin-3. Figure 4D shows that tissue of standard diet fed mice show caveolin-3 staining at the plasma membrane and the T-tubule systems, tissue of MCT diet fed mice has slightly lower overall staining, but caveolin-3 is observed at plasma membranes and the T-tubule system. In contrast, tissue of palmitate diet fed mice has low overall staining and some cardiomyocytes do not show any caveolin-3 protein at the plasma membrane or the T-tubule system. To further investigate the palmitate-induced changes in the intracellular localization of caveolin-3 we exposed HL-1 cardiomyocytes to increasing concentrations of palmitate (Fig. 4E). Cellular membrane systems were separated by sucrose gradient centrifugation and fractions were tested for the presence of caveolin-3 by western blotting. The higher the *in vitro* palmitate concentration was, the more caveolin-3 moved out of the high buoyant fraction (fraction 4, the so called caveolae fraction) to the low buoyant fractions (fractions 8–10), which consist of SR, ER and nuclear membranes.

**Figure 5:**
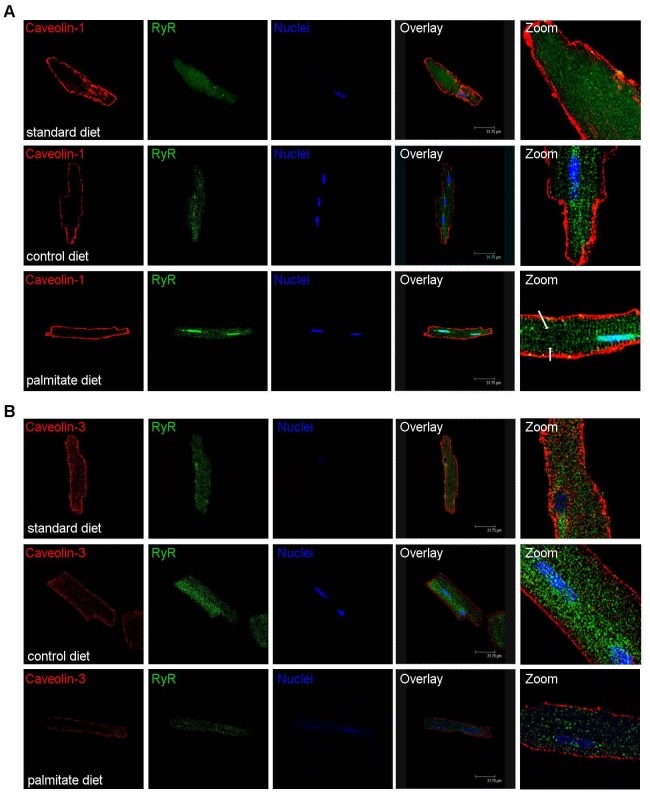
Palmitate-induced loss of T-tubular caveolin-3 and loss of striated appearance of the RyR localization. Intracellular localization of caveolin-1 (**A**) or caveolin-3 (**B**) and RyR in isolated cardiomyocytes from standard, MCT or palmitate diet fed mice. Caveolin-1 and -3 localize to the plasma membrane and the T-tubule system in standard diet fed mice (first row of panels in A and B). In MCT diet fed mice caveolin-1 expression decreases (second row of panels in A) but caveolin-3 expression remains unchanged (second row of panels in B). MCT diet does not change the striated appearance of the RyR (compare both middle panels in A and B). Palmitate diet does not change the expression or localization of caveolin-1 (third row panel A), but expression of caveolin-3 decreases and it is absent from the T-tubule system. In these areas the striated appearance of the RyR is lost (arrows in A and B). All confocal experiments were repeated at least 4 times with similar results.

The loss of caveolin-3 from the T-tubular system suggests a defect in this important membrane signaling system in the heart. Consistent with this idea, caveolin-3 null mice show defects in T-tubule development [35]. Therefore, to test whether palmitate-induced loss of caveolin-3 changes the T-tubule system in the heart, we used isolated cardiomyocytes and stained for ryanodine receptor to visualize the T-tubule system. Figure 5 shows that in standard diet and MCT diet fed mice the T-tubule system is depicted by RyR staining. However, in palmitate diet fed mice there is less RyR staining and in some areas a complete loss of staining. Thus, the diminished contractile performance in the presence of higher extracellular calcium suggests that a defect in the T-tubule system likely coupled with lower RyR expression, which we base on the lower staining in confocal images, cause palmitate – induced contractile dysfunction.

**Figure 6:**
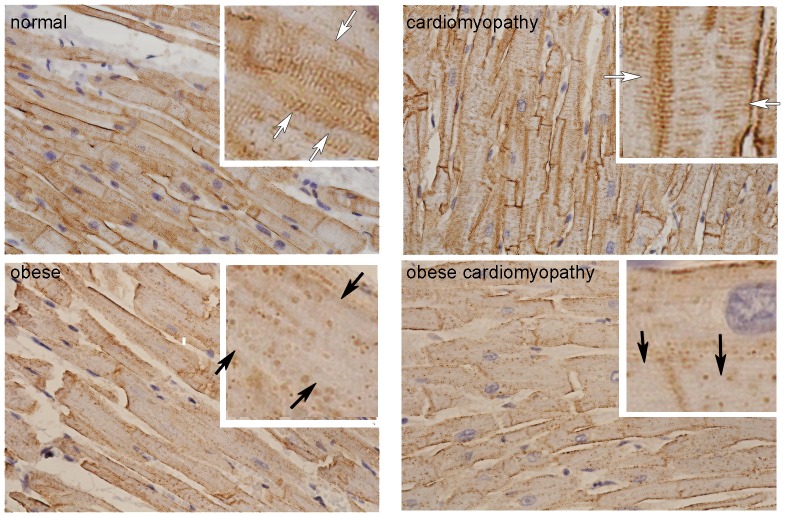
Caveolin-3 expression is decreased in obese human cardiac LV tissue. Representative immunohistochemistry showing caveolin-3 in normal, obese, obese with cardiomyopathy, and non-obese cardiomyopathy human left ventricular cardiac tissue. In normal human cardiomyocytes (upper left panel) caveolin-3 is expressed at the plasma membrane and the T-tubule system (white arrows). In obesity (lower left panel), the expression of caveolin-3 is drastically decreased in the T-tubule system (black arrows), which can occur independent of associated heart disease as the samples with cardiomyopathy (upper right panel) show, but seem to be enhanced in obese cases with cardiomyopathy (lower right panel).

The loss of caveolin-3 in palmitate diet fed mice is consistent with the loss of caveolin-3 in cardiac samples from obese humans (Fig. 6). Although the dietary preferences of these individuals are unknown, the fact that their body mass index was above 30 strongly suggests hyperlipidemia in these individuals. Also there is a clear distinction between cases with non-obese cardiomyopathy and obese cardiomyopathy in the amount of caveolin-3 present in the T-tubule system (white arrows). These data support our findings in mice and support the role of caveolin-3 in lipid induced contractile dysfunction.

### Dietary intervention is sufficient to reverse palmitate-induced caveolin-3 loss

**Figure 7:**
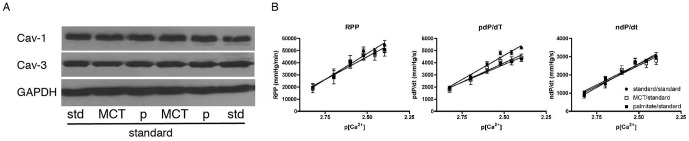
Dietary intervention is sufficient to reverse palmitate-induced caveolin-3 loss. **A**) Western blots of cardiac tissue of mice maintained on standard, MCT, or palmitate diet for 12 weeks followed by 12 weeks of standard laboratory chow feeding. Caveolin-1 and -3 expression is normal. (Std standard, MCT medium chain triglyceride high fat control diet, P palmitate). **B**) Ex vivo contractile performance (RPP, pdP/dt and ndP/dt) and calcium sensitivity normalizes after 12 weeks of standard laboratory chow feeding. N = 6 (3 male and 3 female) per diet, linear regression analysis is not significant.

To find out whether the lipid-induced changes in cardiac membranes and the loss of caveolin-3 is reversible, we changed the high fat diet fed mice after 12 weeks to normal laboratory chow for another 12 weeks. Western blotting confirmed that caveolin-1 and caveolin-3 levels normalize (Fig. 7A) and contractile performance and calcium sensitivity in the isolated heart was also normal (Fig. 7B), showing no significant differences between standard, MCT, and palmitate diets at this time point.

## Discussion

Obesity is a growing epidemic in the Western world and understanding mechanisms of the associated cardiovascular diseases is imperative for developing treatment strategies. Here we present a new mechanism for lipid-induced cardiac contractile dysfunction that resembles the cardiomyopathy observed in caveolin-3 loss-of-function mice [25]. We present evidence that high intake of dietary palmitate disturbs sphingolipid homeostasis, which leads to contractile dysfunction by inducing the loss of caveolin-3 from the T-tubule membrane system. Our findings are further supported by immunohistochemistry findings in human cardiac tissue samples of non-obese and obese humans, in which the obese tissue samples showed a drastic reduction of caveolin-3 expression in cardiomyocytes.

### Palmitate-induced contractile dysfunction is independent of mechanisms causing lipid-induced diabetic cardiomyopathy

Previous studies used high fat feeding to induce insulin resistance and diabetes. For example, Kim et al. [36] compared 10% and 60% fat content in rodent diets and found insulin resistance and diabetes after 8 weeks in mice fed the 60% fat diet. Similarly, Lessard et al. [37] used 58% fat content in rats to induce insulin resistance in 4 weeks. In contrast to these studies, the time line of this high-fat feeding study and the lipid content was chosen in such a way that mice were not insulin resistant or diabetic. After 12 weeks on either the MCT or the palmitate diet mice were not hyperglycemic, hypertriglyceridemic or hypercholesterolemic. Only the palmitate diet-fed group had slight hyperinsulinemia with an onset at 10 weeks of palmitate diet feeding. Thus, our work suggests that contractile dysfunction and diabetic cardiac changes are, under our conditions, independent and uncoupled events.

### Sphingolipid accumulation disturbs calcium regulation and causes systolic contractile dysfunction

The exposure to palmitate and subsequent accumulation of ceramide has long been considered detrimental to tissues due to its activation of apoptotic effectors, including JNK [14], mitochondrial cytochrome C release [38], Bax expression [39], and caspase 3 activation. To further investigate the mechanism of ceramide on cells, many studies used synthetic short chain ceramides that are easily taken up by the cells and are subsequently metabolized. However, these short chain ceramides may change the biophysical properties of the membrane in a different way than long-chain ceramides do [40], as they cause less disturbances in the lipid bilayer formation. To overcome this concern, we used a different, more biologically relevant approach to increase cardiac tissue levels of ceramides and sphingomyelins. Feeding a palmitate rich diet led to the accumulation of medium and long-chain ceramides and sphingomyelins, which were incorporated into cellular membranes. Other studies have shown that palmitate exposure induces apoptosis in cardiomyocytes [14], [41], [42]. To determine whether our approach of dietary palmitate induces apoptosis, we tested for caspase-3 activation and performed TUNEL staining in histological sections of cardiac tissue. We did not observe activation of caspase-3 in the palmitate diet fed mice. More over, TUNEL staining of cardiac tissue was negative as well (data not shown). This result suggests that dietary delivery of palmitate is different from direct palmitate exposure *in vitro* and provides the advantage of not inducing apoptosis in the heart. Thus, we conclude that the contractile dysfunction we observed in the palmitate diet fed mice is primarily associated with the inability of the hearts to regulate calcium levels.

### Palmitate-induced loss of membrane caveolin-3 as a new mechanism for lipid-induced cardiomyopathy

At least 30 inactivating mutations have been identified in the human gene coding for caveolin-3, that lead to the loss of caveolae in skeletal muscle with four major associated disease complexes: limb-girdle muscular dystrophy, rippling muscle disease, hyperCKemia, and distal myopathy [43]–[46]. These diseases are mainly autosomal-dominant, and patients present with a broad range of symptoms that overlap between the four diseases. In the heart, caveolin-3 mutations are associated with hypertrophic and dilated cardiomyopathy [47] and long-QT syndrome [48]. In addition, caveolin-3 mutations seem important in some cases of sudden infant death syndrome [49]. All mutations result in decreased caveolin-3 and caveolae in muscle cells. The loss of caveolin-3 in the mouse reduces the number of caveolae in muscle and induces T-tubule abnormalities and a phenotype similar to muscular dystrophy [35]. Caveolin-3 null mice also develop cardiac hypertrophy that leads to dilated cardiomyopathy [25], [50]. Our study shows that palmitate-induced loss of caveolin-3 is associated with a contractile dysfunction phenotype that is similar to the cardiomyopathy reported in the caveolin-3 null mice [35]. To determine the underlying defect in calcium regulation, we determined expression of the most important calcium regulating proteins in the heart, SERCA, DHPR, and the ryanodine receptor. SERCA and DHPR levels were unchanged in hearts from palmitate diet-fed mice, consistent with an unchanged diastolic contractile function. In contrast to SERCA, the RyR was downregulated in cardiomyocytes from palmitate diet-fed mice (Fig. 5). The decrease and mislocalization of RyR protein is consistent with the loss of T-tubule structure, which is expected to cause impaired calcium handling and contractile dysfunction. Recently, the direct binding of the dihydropyridine receptor and caveolin-3 was reported in skeletal muscle [51]. The dihydropyridine receptor is the partner of the RyR and responsible for the inflow of the trigger calcium that activates the RyR. We suggest that the loss of caveolin-3 disturbs the close interaction between the dihydropyridine receptor and the RyR, interfering with the calcium-induced calcium release. Importantly, we show that dietary intervention is sufficient to reverse the palmitate-induced caveolin-3 loss. This reversibility indicates that caveolin-3 loss is regulated by dietary lipid intake, and that changes in membrane lipids can be reversed. This finding of reversibility has high translational significance, as simple dietary intervention may be sufficient to prevent and reverse the progression of the cardiomyopathic phenotype that develops in a subset of obese individuals.

Recently, the loss of T-tubule structure and organization was found to be a common theme for heart failure of different etiologies and in different species [52]–[55]. In particular, the work by Wei et al. [55] shows that the degree of heart failure is linked to the degree of T-tubule loss. The importance of this membrane system for cardiac contractile performance is further supported by the binding of beta adrenergic receptors to caveolins [56] and the work by Nikolaev et al. [54] who showed that in heart failure beta2 adrenergic receptors, which are found in the T-tubule system, re-localize to plasma membrane areas essentially eliminating the distinction of beta1 and beta2 adrenergic responses in the failing heart. Based on these studies and the fact that caveolin-3 is required for normal T-tubule formation during cardiac development [20], [35], because caveolin-3 is a crucial and important scaffolding protein that enables compartmentalization of cellular signaling, we suggest that the expression and intracellular localization of caveolin-3 in heart failure should be investigated as a potential therapeutic target for the improvement of contractile function. This view is further supported by the significant loss of caveolin-3 in heart samples of obese humans, which can be observed prior to other morphological changes that are associated with cardiomyopathy and heart failure.
